# How to Render Cow’s Milk a More Suitable Food for Children

**Published:** 1858-11

**Authors:** 


*How to render Cow's Milk a more Suitable Food for Children.* By Dr. Gumprecht, of Hamburgh.

Dr. Gumprecht prefaces his observations by remarking upon the fact that milk often disagrees with children, producing indigestion, acidity, flatulence, colic, diarrhœa, &c. &c. In consequence of this, it has been proposed to improve it by the addition of water and sugar of milk, which experience has proved to have imperfectly attained the object in view. Reflecting on the effect of salt in rendering the food for adults not only more palatable, but also more digestible, increasing the activity of the glands of digestion, and rendering the albuminous substance and fat soluble in the fluids of the stomach, Dr. Gumprecht was led to the idea of adding salt to milk, both for weaned and older children, with the result of not only preventing the derangement of digestion, but moreover of removing them in cases where they previously existed. No author who has written on the nutriment of weaned children has spoken of this most useful addition to milk; but a Dutch physician mentioned to Dr. Gumprecht, in conversation, that in his practice in Holland he had frequently added a little salt to milk for weaned children with most satisfactory consequences.

In the rural districts of Holland, salt is frequently added to the fodder for pigs and cattle, for the purpose of preventing diarrhœa, which so often exists in consequence of imperfect digestion, and this suggested the adding salt to milk, not merely for healthy children, but for strumous children and such as are affected with worms. Dr. Gumprecht quotes a passage from L. Nussdorff's "Lehrbuch der Gesundheitspflege," 1856, on the importance of salt in the nutriment of man and animals.

With regard to the quantity of salt which should be added to the milk, it must depend on the age of the child. To render cows' milk like human milk, it should be boiled and skimmed, and a little sugar of milk and salt added.—*Journal für Kinderkrankheiten*, and *Dublin Hospital Gaz.*, from *Ranking's Abstract of Med. Sciences.*

